# Pyoderma Gangrenosum Post Reduction Mammoplasty: An Unusual Postoperative Complication

**DOI:** 10.7759/cureus.77792

**Published:** 2025-01-21

**Authors:** Grecia Mariana Cantú-Fonseca, Ilse Marilu Gutiérrez-Villarreal, Monica Patricia Ceballos-Pérez, Sandra Carolina Méndez-Sosa, Circe Ancona-Castro

**Affiliations:** 1 Dermatology, Hospital Regional Monterrey, Instituto de Seguridad y Servicios Sociales de los Trabajadores del Estado (ISSSTE), Monterrey, MEX; 2 Internal Medicine, Hospital Regional Monterrey, Instituto de Seguridad y Servicios Sociales de los Trabajadores del Estado (ISSSTE), Monterrey, MEX

**Keywords:** autoimmune disease, post surgery complication, pyoderma gangrenosum, pyoderma gangrenosum after breast reduction, treatment choices

## Abstract

We present a case of a patient with pyoderma gangrenosum, a rare complication associated with surgical procedures. Breast reduction is the second most common etiology within this category. This case emphasizes the importance of early diagnosis to start therapy on time to stop the inflammatory process and prevent its progression, achieving adequate re-epithelialization, as well as emphasizes the use of an immunomodulator with steroid treatment to offer better results that impact the quality of life positively.

## Introduction

Pyoderma gangrenosum (PG) is a rare neutrophilic dermatosis with a worldwide incidence of three to 10 cases per million habitants. It can appear at any age, but it is more frequent in people between 20 and 50 years of age, with no gender predilection [1,2]. Its etiopathogenesis involves the dysregulation of the innate and adaptive immune systems, deriving an autoinflammatory process with high levels of tumor necrosis factor-alpha and interleukins 1, 8, 12, 17, 23, and 36 [2].

There are six different variants of PG described, which include bullous, pustular, ulcerative, vegetative, atypical bullous, and post-surgical variants [3]. It is mostly related to systemic diseases, but in up to 25% of the patients, surgical antecedent has been identified as a cause [1]. Post-surgical pyoderma gangrenosum (PSPG) can be suspected if there is a rapid deterioration of the wound after a median of seven postoperative days, including necrotizing tissue and associated pain that does not improve with antibiotic therapy [1,3].

So far, there is no standardized treatment for PG, but therapy with corticosteroids, topical or systemic, depending on the severity and the use of immunosuppressants, has been reported to be effective for resolution in these patients [3,4].

This case highlights the need for a high index of suspicion to obtain early treatment to avoid bigger complications.

## Case presentation

The patient was a 39-year-old female with only a surgical history of rhinoplasty and liposculpture without complications. Her condition began four days after the surgical procedure of breast reduction. She presented with changes in skin color, papules, and vesicles with a consequent presence of purulent material, evolving to painful ulcers over the course of 48 hours with rapid growth. Due to a torpid evolution without improvement with antibiotics and a low dose of oral corticosteroid treatment previously given by her surgeon, she was referred to the medical institution 40 days after the surgical procedure. In the hospital, she underwent surgical debridement with a previous sampling for bacterial culture to rule out resistance to antibiotics and to exclude different etiologies of probable fungal infection. After that, an assessment at the service of dermatology was requested.

**Figure 1:**
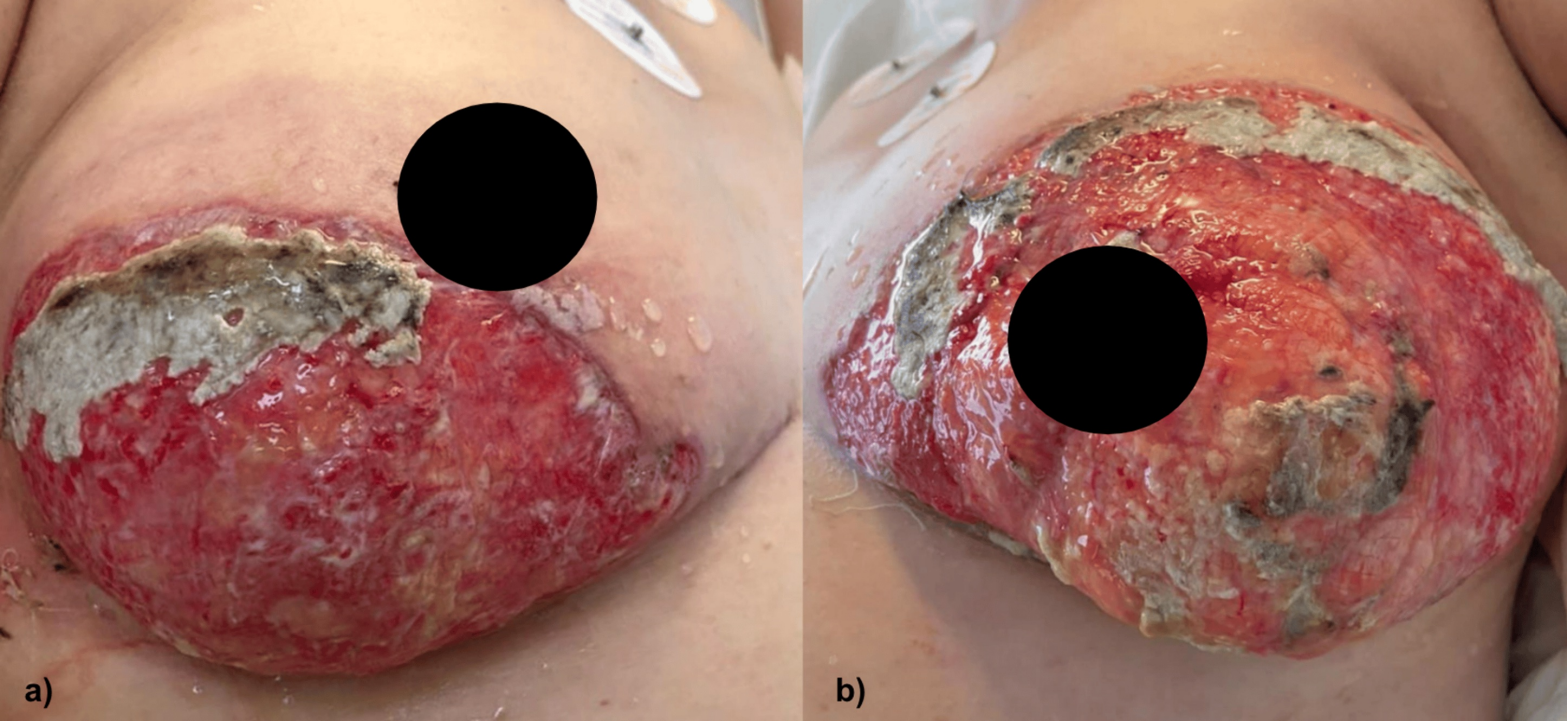
Dermatosis localized to the trunk, affecting (a) right and (b) left mammary regions with the presence of ulcers with fibrin tissue and serohematic exudate 40 days after the surgical procedure.

On admission, there was evidence of dermatosis localized to the trunk affecting the bilateral mammary region, asymmetrically, characterized by two ulcers, the right one with a diameter of 10 cm and the left one of 12 cm, with granulomatous background, covered by serohematic exudate, areas with bedsores and fibrin, and surrounded by an elevated, violaceous, irregular, well-defined border (Figure 1).

**Figure 2:**
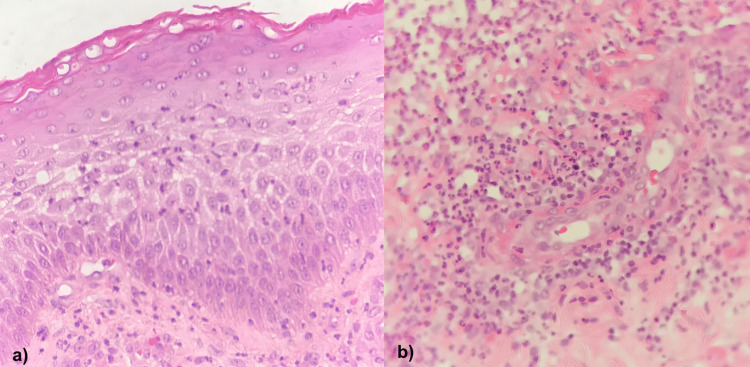
Punch skin biopsy of dermatosis of the left breast showing (a) epidermis with polymorphonuclear (PMN) exocytosis and (b) dermis with mixed inflammatory infiltrate of lymphocytes and PMN with perivascular predominance, as well as marked leukocytoclastic vasculitis with erythrocyte extravasation.

Because of its evolution, a punch skin biopsy of the dermatosis was done, with compatible data of PG (Figure 2). A tissue culture was reported as negative.

She underwent multidisciplinary management with high-dose oral corticosteroids (1 mg/kg/day) on subsequent tapering without the expected improvement, so oral cyclosporine (4 mg/kg/day) and topical application of calcineurin inhibitor were added. Also, measures with pirfenidone, blotting powders, and petroleum jelly were added to accelerate re-epithelialization.

**Figure 3:**
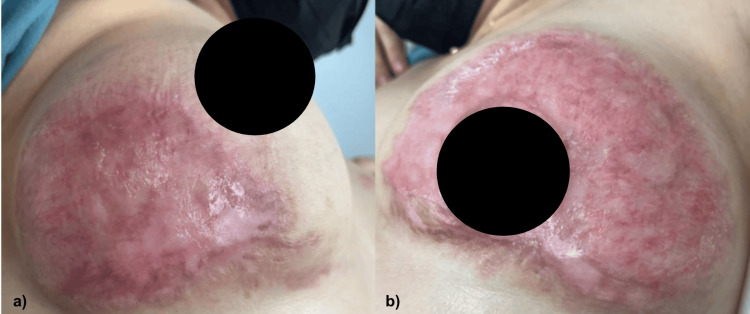
Decrease in the size of ulcers of (a) right and (b) left breasts, with adequate cicatrization and without the presence of lesions with loss of the skin continuity solution.

During her 15 days of hospitalization, she remained hemodynamically stable, without complications, showing improvement after seven days with combined therapy. Because of her adequate evolution, her follow-up was continued as an outpatient. Currently, 10 months later, the patient is showing adequate signs of re-epithelialization and continues with the absence of the disease activity (Figure 3).

## Discussion

PG is a rare pathology associated with high morbidity when treatment is not initiated in a timely manner. Although the pathogenesis has not been well established, it is thought that it is caused by the loss of the immunological privilege of the follicular unit. The literature described mostly the association with systemic diseases and other few cases in relationship with surgical procedures happening at sites of cutaneous trauma [2,5].

A systematic review of multiple cases by Ehrl et al. and Zuo et al. reported that breast reduction is the surgical procedure that most often triggers PSPG, confusing it with postsurgical infection in almost all cases, leading to a delay in the diagnosis process and, consequently, in the start of its treatment [2,3].

The major criterion is exposed (a) neutrophilic infiltrate in the biopsy, and minor criteria include (a) exclusion of infection, (b) patergia phenomenon, (c) history of inflammatory bowel disease, (d) history of papule, vesicle, or pustule that ulcerates within four days, (e) peripheral erythema, undermined border, or pain at the site of ulceration, (f) multiple ulcerations and at least one affecting lower extremities, (g) cribriform scar, and (h) a decrease in ulcers one month following the use of immunosuppressants. For the diagnosis of this disease in any of its variants, the major criteria and at least two minor criteria are necessary [1,4,6].

In this case, the clinical picture was aggravated due to the pathergy phenomenon, secondary to the treatment established with debridement, leading to a larger area of inflammation, so unnecessary debridement needs to be avoided in suspicious cases. Her areola-nipple complexes appeared to be mostly spared, probably because of the pathogenesis of this pathology and the type of tissue involved. This patient was refractory to initial treatment with systemic steroids, requiring the addition of an immunomodulator to stop the progression of the injuries, presenting an adequate response after that.

It is established that steroids can be used as a first-line treatment; however, in case of refractoriness, another immunomodulator agent can be added to help remit the clinical picture. In some cases, biological medications need to be considered. The response varies among patients, so at the moment, there is no standardized treatment for the disease [3,7]. During the inflammatory and healing phases, it is crucial to optimize moisture balance and prevent infection, so wound dressings can be used to achieve improvement [8].

The key to good management is the early suspicion of a post-surgical wound that presents a rapid progression and does not improve with conventional treatment, as reported in the different cases in the literature [2,3,5].

## Conclusions

This variety of PG implies a diagnostic challenge; there are no rapid and specific tests, so early suspicion is important to initiate timely treatment and avoid major complications and morbidity. The studies are limited due to the low incidence, so it should be further explored for new knowledge. We emphasize the use of oral steroids and immunomodulators for its management. A greater understanding of its molecular foundations will allow the development of specific treatments.
